# Salivary biomarkers in burning mouth syndrome: an umbrella review of systematic reviews

**DOI:** 10.1186/s12903-026-08585-z

**Published:** 2026-05-20

**Authors:** Mariana Vallera Machete, Anis Bouaita, Andrés Blanco-Carrión, José López López, Carmen Martín Carreras-Presas, Paulo Mascarenhas, José Grillo Evangelista, Pedro Ferreira Trancoso, António Mano Azul, Carlos Zagalo

**Affiliations:** 1 Egas Moniz School of Health & Science, Campus Universitário, Quinta da Granja, Monte de Caparica, Caparica, 2829-511 Portugal; 2 Egas Moniz Center for Interdisciplinary Research (CiiEM), Egas Moniz School of Health & Science, Campus Universitário, Quinta da Granja, Caparica, Almada, 2829-511 Portugal; 3 Universidad de Santiago de Compostela, Praza do Obradoiro, s/n 15705 Santiago de Compostela, A Coruña, España; 4 Facultad de Medicina y Ciencias de la Salud, Odontología, Universitat de Barcelona (UB), Campus de Medicina - Clínic August Pi i Sunyer. Casanova, 143, Barcelona, 08036 España; 5 Dentistry Department, Faculty of Health and Biomedical Sciences, Universidad Europea de Madrid, C. Tajo, s/n, Villaviciosa de Odón, Madrid, 28670 España

**Keywords:** Burning mouth syndrome, Salivary biomarkers, Cortisol, Alpha-amylase, Orofacial pain, Umbrella review

## Abstract

**Background:**

Burning Mouth Syndrome (BMS) is a chronic orofacial pain condition without objective diagnostic criteria and with a substantial impact on quality of life. This umbrella review aimed to evaluate the available evidence on salivary biomarkers in BMS, identify which biomarkers show the most consistent associations with the condition, and assess the methodological quality of existing systematic reviews and meta-analyses.

**Methods:**

A comprehensive search of PubMed, Embase, Web of Science, Scopus, TRIP, and Google Scholar was conducted without language or date restrictions. Systematic reviews and meta-analyses assessing salivary biomarkers in patients with BMS were included. Of the 561 records screened, six reviews met the eligibility criteria. Methodological quality was assessed using the AMSTAR-2 tool.

**Results:**

AMSTAR 2 appraisal rated four reviews as moderate confidence and two as low confidence; none was rated high or critically low. The evidence base showed very high overlap among primary studies, indicating that repeated findings across reviews should not be interpreted as fully independent replications. Cortisol showed the most consistent evidence of elevation in BMS. α-amylase and IgA were also frequently elevated, particularly in reviews on stress-related biomarkers. However, their interpretation was limited by methodological heterogeneity, overlap of primary studies and limited disease specificity. Cytokines, sex hormones, opiorphin, trace elements and oxidative stress-related markers showed inconsistent or sparse evidence.

**Conclusions:**

Salivary cortisol, α-amylase and IgA may reflect stress-related neuroendocrine, autonomic and mucosal immune dysregulation in BMS. However, current evidence remains methodologically limited and does not support the use of any salivary biomarker as a standalone diagnostic test.

**Clinical relevance:**

Saliva-based biomarkers may support mechanistic phenotyping in future research, but standardised protocols, rigorous study designs and improved patient stratification are required before they can inform clinical decision-making.

**Supplementary Information:**

The online version contains supplementary material available at 10.1186/s12903-026-08585-z.

## Introduction

Burning mouth syndrome (BMS) represents one of the most enigmatic and frustrating challenges in modern oral medicine. Characterized by a chronic, burning sensation of the oral mucosa without visible clinical lesions or detectable systemic causes, BMS primarily affects middle-aged women, with a prevalence estimated at 0.7–15% in the general population [1–3]. The condition significantly impairs quality of life, often leading to anxiety, depression, and sleep disturbances. Despite its burden, BMS remains a “diagnosis of exclusion,” a label that frequently subjects patients to prolonged diagnostic odysseys, unnecessary treatments, and the invalidating stigma of having their pain dismissed as psychogenic [4–8].

Over the past decade, the understanding of BMS has shifted fundamentally from a purely psychological model to one of complex neuropathic and neuroendocrine dysregulation. Current hypotheses implicate peripheral small-fiber neuropathy, central sensitization, and altered dopaminergic control, often exacerbated by chronic stress and gonadal hormone depletion [9–11]. This paradigm shift in physiology has driven an intense search for objective biomarkers to validate patients’ subjective experiences and elucidate underlying mechanisms. Among biological fluids, saliva has emerged as the primary candidate for this purpose. As a “liquid biopsy,” saliva offers a non-invasive, cost-effective window into the body’s neuroendocrine and immune status, reflecting systemic changes in cortisol, cytokines, and oxidative stress markers that mirror the pathophysiology of chronic pain [12–16].

However, the rapid proliferation of biomarker research has created a chaotic evidence landscape. While numerous individual studies have proposed diverse candidates—ranging from inflammatory cytokines such as IL-6 to stress hormones such as cortisol—the results have been inconsistent and often contradictory. Recently, several systematic reviews and meta-analyses have attempted to synthesize this data, yet they too vary in their scope, inclusion criteria, and conclusions [17–21]. Some identify cortisol as a definitive marker, while others highlight methodological flaws that preclude firm recommendations. This fragmentation leaves clinicians and researchers without a clear consensus: Which biomarkers are reliable and which are merely statistical noise?​.

To address this uncertainty, a higher level of evidence synthesis is required. This article presents an umbrella review (a systematic review of systematic reviews) designed to critically appraise and consolidate the existing body of evidence on salivary biomarkers in BMS. By evaluating the methodological quality of previous syntheses and triangulating their findings, this review aims to distinguish robust biological signals from methodological artifacts. In doing so, we seek to provide a definitive “state of the science” that identifies the most promising biomarkers for clinical translation and charts a rigorous path for future research.

## Materials and methods

### Review design, protocol and reporting framework

This umbrella review synthesized evidence from systematic reviews and meta-analyses on salivary biomarkers in patients with Burning Mouth Syndrome (BMS). The review was reported in accordance with the PRISMA 2020 statement [22] and, where applicable, methodological principles for overviews of reviews. No prospective protocol was registered. To improve transparency and reproducibility, the eligibility criteria, search strategy, study selection procedures, data extraction framework, methodological appraisal approach and synthesis methods are described in detail below and provided in full in the Supplementary Material.

### Focused question and eligibility framework

The focused question was structured using the PICO framework: in patients with BMS, which salivary biomarkers differ from those of individuals without BMS, and what is the consistency and methodological robustness of this evidence across existing systematic reviews and meta-analyses? The population was defined as patients diagnosed with BMS; the index exposure was the measurement of salivary biomarkers; the comparator was a control group without BMS; and the outcome was the direction, consistency and potential clinical relevance of differences in salivary biomarker levels between BMS and control groups.

### Information sources and search strategy

A comprehensive literature search was conducted in PubMed/MEDLINE, Scopus, Web of Science, Embase, TRIP Database and Google Scholar to identify systematic reviews and meta-analyses reporting salivary biomarker findings in BMS. The search combined controlled vocabulary and free-text terms related to BMS, saliva, salivary biomarkers and evidence synthesis. Search terms were adapted to each database’s syntax, and the complete database-specific strategies are provided in Supplementary Table S1. Google Scholar and TRIP were used as complementary search sources to increase sensitivity and identify potentially relevant evidence syntheses not retrieved by bibliographic databases. Google Scholar screening was limited to the first 200 records sorted by relevance, as specified in Supplementary Table S1. No separate grey-literature databases, beyond those sources, were searched for the umbrella review. The search was designed to be sensitive enough to identify reviews that embed salivary biomarker data within broader biomarker syntheses. However, eligibility, data extraction and synthesis were restricted to salivary biomarker evidence. Reviews or review sections reporting only serum, plasma, blood, urine or tear biomarkers were not included in the evidence synthesis.

### Eligibility criteria

Systematic reviews, with or without meta-analysis, were eligible if they met all of the following criteria: (1) included human studies involving patients diagnosed with BMS; (2) assessed at least one salivary biomarker in BMS patients; (3) included a comparator group without BMS or synthesised primary studies that included such comparisons; (4) reported extractable salivary biomarker findings, either narratively or quantitatively; and (5) provided sufficient methodological detail to allow appraisal of the review process. Reviews were excluded if they: (1) were narrative reviews, scoping reviews without systematic methods, editorials, letters, conference abstracts or primary studies; (2) assessed only serum, plasma, blood, urine, tear, genetic or tissue biomarkers without extractable salivary biomarker data; (3) focused exclusively on treatment effects without baseline salivary biomarker comparisons between BMS and controls; (4) did not report BMS-specific biomarker findings; or (5) did not provide sufficient information to support data extraction or methodological appraisal.

### Study selection

After duplicate removal, two reviewers independently screened titles and abstracts against the predefined eligibility criteria. Potentially eligible reports were then assessed in full text by the same reviewers. Disagreements at either stage were resolved by discussion and, when consensus could not be reached, by consultation with a third reviewer. Reference lists of eligible reviews were hand-searched to identify additional relevant publications.

### Data extraction

Data were extracted using a structured extraction form. For each included review, the following information was recorded: first author, year of publication, review type, databases searched, search period, number and type of included primary studies, BMS diagnostic criteria reported in the primary studies, number of BMS and control participants when available, salivary biomarkers assessed, type of saliva analysed, analytical methods, quantitative or narrative synthesis methods, main salivary biomarker findings, direction of association, statistical significance, heterogeneity, certainty or quality assessment methods used by the review authors, and review conclusions.

### Methodological appraisal of included reviews

The methodological quality of the included reviews and confidence in their findings were appraised using AMSTAR 2. AMSTAR 2 was not treated as a numerical scoring system or as a conventional primary-study risk-of-bias tool. Instead, each review was classified according to the AMSTAR 2 guidance as high, moderate, low or critically low confidence, based on the presence of critical and non-critical weaknesses. Particular attention was given to protocol registration, adequacy of the literature search, justification for the exclusion of studies, assessment and consideration of risk of bias in the primary studies, appropriateness of meta-analytical methods, assessment of publication bias and reporting of conflicts of interest. Domain-level judgments and overall confidence ratings are presented in Supplementary Table S2.

### Assessment of overlap across primary studies

Because this was an umbrella review, overlap of primary studies across the included reviews was assessed to avoid interpreting repeated inclusion of the same primary evidence as independent confirmation. A citation matrix was constructed in which rows represented unique primary studies and columns represented included reviews. For each review, the presence or absence of each primary study was recorded. Where sufficient information was available, the corrected covered area (CCA) was calculated to estimate the degree of overlap across reviews. The CCA was calculated as: CCA = (N − r) / (r × c − r), where N is the total number of primary-study occurrences across reviews, r is the number of unique primary studies, and c is the number of included reviews. Overlap was interpreted as slight, moderate, high or very high according to established CCA thresholds. When relevant, overlap was also considered pairwise between reviews. Findings from highly overlapping reviews were interpreted cautiously, with greater weight given to the most recent, comprehensive and methodologically robust review.

### Data synthesis and interpretative framework

A narrative synthesis was performed because the included reviews differed in scope, eligibility criteria, primary studies included, biomarker categories, saliva collection procedures, analytical methods and statistical approaches. For reviews that included both salivary and non-salivary biomarkers, only salivary biomarker data were extracted. Serum, plasma, blood, urine, tear and tissue biomarker results were not included in the synthesis and were not used to support the conclusions of this umbrella review.

No new pooled meta-analysis was conducted at the umbrella-review level. Instead, findings were synthesized by biomarker family: (1) stress-related and neuroendocrine markers, including cortisol, α-amylase and IgA; (2) inflammatory cytokines and immune mediators; (3) sex hormones and related endocrine markers; (4) pain-related biomarkers, including opiorphin and neuropeptides; and (5) trace elements, minerals and oxidative stress-related markers.

For each biomarker or biomarker family, the synthesis considered: the number of reviews reporting the marker, whether findings were based on meta-analysis or narrative synthesis, the direction of association between BMS and controls, consistency across reviews, overlap of primary studies, methodological quality of the reviews, heterogeneity in saliva collection or assay procedures, and the extent to which the biomarker could be interpreted as disease-specific, mechanistic, stress-related or exploratory.

## Results

### Study selection

**Fig. 1:**
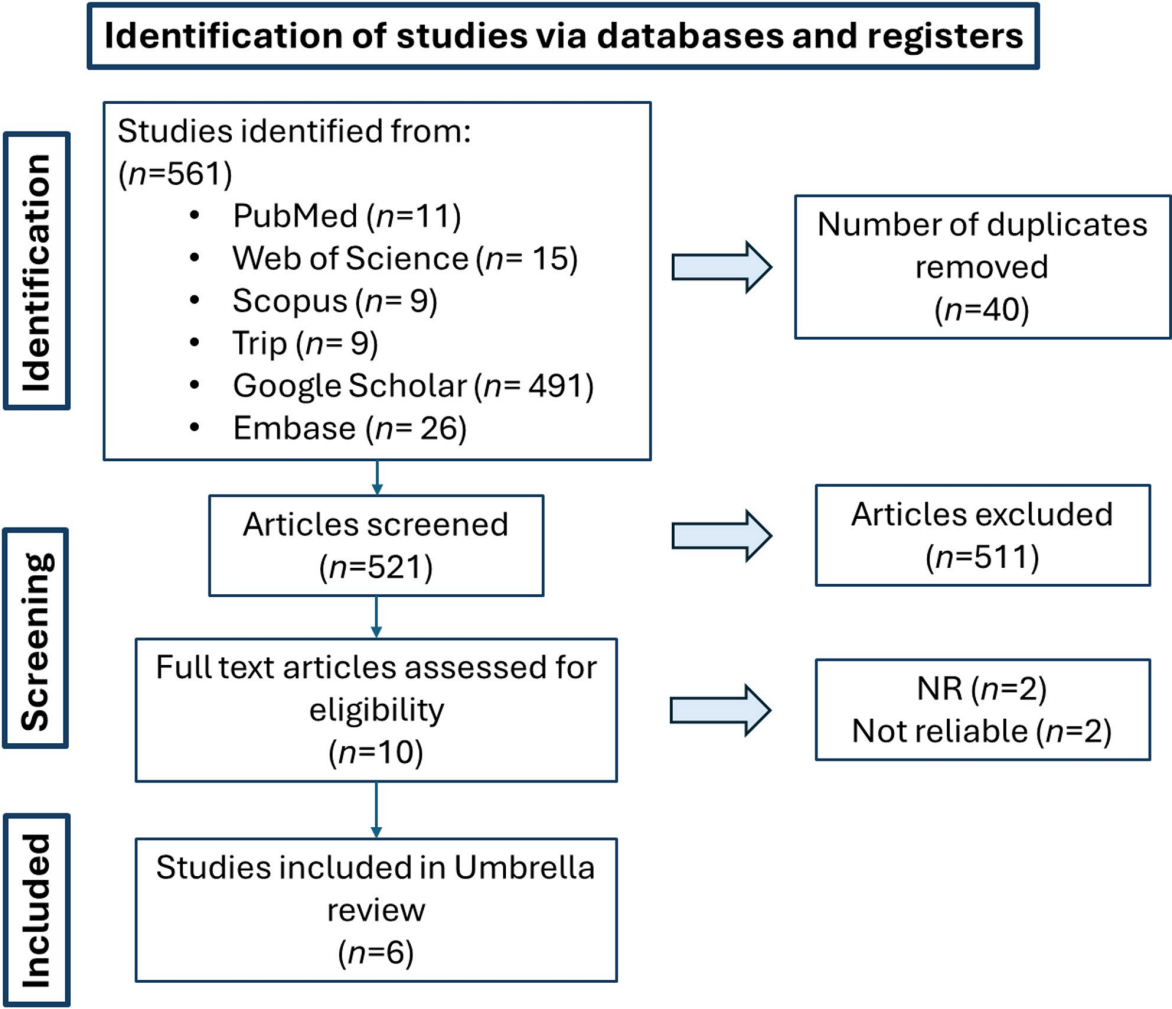
PRISMA flow-chart diagram

The PRISMA flow diagram (Fig. 1) sets out the entire literature search process. The database search identified 561 records. After removing 40 duplicates, 521 records were screened based on title and abstract. Of these, 511 were excluded for not meeting the eligibility criteria, leaving 10 reports for full-text assessment. Four reports were excluded after full-text review for the reasons detailed in Table 1. Six systematic reviews, with or without meta-analysis, were included in the final umbrella review.

Because the scope of the review was restricted to salivary biomarkers, reviews that also reported serum, plasma, blood, urine or tear biomarkers were retained only when salivary biomarker data were extractable. Non-salivary biomarker findings were not included in the evidence synthesis.

**Table 1:** Reasons for excluding four full-text articles

No.	References	Rationale
1	Porporatti AL, de Oliveira Machado CA, Alajbeg I, Alajbeg IZ, Paszynska E, Dmitrzak-Weglarz M, et al. Opiorphin as a biomarker of orofacial conditions: A meta-analysis. Sci Rep. 2023;13(1):15,533. doi:10.1038/s41598-023-42051-y	Not eligible: the review focused on opiorphin across heterogeneous orofacial conditions and biological fluids; BMS was only one subgroup, and the review question was not aligned with the present BMS-focused salivary biomarker umbrella review.
2	Campello CP, Pellizzer EP, Vasconcelos BCDE, Moraes SLD, Lemos CAA, Muniz MTC. Evaluation of IL-6 levels and + 3954 polymorphism of IL-1β in burning mouth syndrome: A systematic review and meta-analysis. J Oral Pathol Med. 2020;49(10):961–968. doi:10.1111/jop.13018	Not eligible: the review included genetic polymorphism data and was not restricted to extractable salivary biomarker evidence aligned with the present review question.
3	Pakravan F, Chatraei F, Heidari Z, Nilchian F, Ghazavi R, Nasr Isfahani M. Evaluation of the relation between salivary alpha-amylase level and oral diseases with stress etiology: A systematic review and meta-analysis. Res Sq [Preprint]. 2023. doi:10.21203/rs.3.rs-3318020/v1	Not eligible: not BMS-focused; insufficient methodological reporting and/or appraisal reliability for inclusion in the umbrella review.
4	Eslami H, Azad S, Shafiee-Kandjani AR, Fakhrzadeh V, Tavakoli F. Investigation of salivary biomarkers IL-6, IL-1β, and TNF-α in burning mouth syndrome: A systematic review. Middle East J Rehabil Health Stud. 2022; 10:e131734. doi:10.5812/mejrh-131734	Not eligible: insufficient methodological reporting and/or appraisal reliability for inclusion in the umbrella review.

### Characteristics of included reviews

Finally, six systematic reviews were included in the current umbrella review [17, 18, 20, 21, 23, 24]. Two included studies are systematic reviews without meta-analysis [17, 24] and the other four are systematic reviews with meta-analysis [18, 20, 21, 23]. The main characteristics of these reviews are displayed in Table 2. The included reviews were published between 2019 and 2024 and differed substantially in scope, eligibility criteria, biomarker focus and statistical approach. Two reviews focused broadly on salivary biomarkers in BMS; two addressed specific biomarker families, namely cytokines and sex hormones; one examined salivary and serum biomarkers in relation to psychological disorders; and one evaluated the association between BMS, stress and stress-related biomarkers. The number of primary studies included in the reviews ranged from 4 to 30 in qualitative syntheses, with quantitative syntheses performed only for selected biomarkers. The most frequently synthesized salivary markers were cortisol, α-amylase, IgA, DHEA, opiorphin, selected cytokines, and trace elements or minerals. However, the ability to compare findings across reviews was limited by heterogeneity in BMS diagnostic criteria, saliva collection procedures, saliva collection mode (stimulated versus unstimulated), timing of collection, assay methods, reporting units and adjustment for psychological or hormonal confounders.

To provide a structured comparison of the salivary biomarker evidence, findings were grouped by biomarker family and summarised according to direction of association, number of contributing reviews, consistency, main methodological limitations and clinical interpretation (Table 3). This synthesis was restricted to salivary biomarkers, even when the source reviews also reported non-salivary biomarkers.

**Table 2:** Characteristics of selected systematic reviews

Author	Analysis Method	Databases	Location	Research period	Number of articles
Fernández-Agra 2022 Ref. [21]	SR-MA	PubMed/MEDLINE, Scopus, Web of Science, Cochrane Library	Spain (University of Madrid)	Until July 5th, 2022	17 studies
Kappes et al., 2023 Ref. [18]	SR-MA	PubMed, Web of Science, Cochrane Library	France (CHU Clermont-Ferrand)	Until September 2022	− 29 studies in qualitative assessment − 15 meta-analysis
Kishore et al., 2019 Ref. [17]	SR	PubMed/MEDLINE, Embase	Pakistan (Ziauddin University, Karachi)	From November 1986 until November, 2018	8 studies
He et al., 2024	SR-MA	PubMed, Embase, Cochrane Library	China (Central Hospital of the Longhua District, Shenzhen and Medical University of Southwest, Luzhou)	Until March 15th, 2023	12 studies
Ref. [20]					
Porporatti et al., 2023 Ref. [23]	SR-MA	EMBASE, LIVIVO, PubMed, Scopus, Web of Science, Google Scholar, Open Grey, ProQuest, Clinical trial.	Université Paris Cité (France) and Brazil’s NARSM (Systematic Review & Meta-Analysis Center at Tuiuti University)	Until January 12th, 2022	30 studies
Brauwers et al., 2024 Ref. [24]	SR	PubMed, Scopus, Web of Science, Cochrane Library, Embase	Brazil	Until October 2022	4 studies

**Author**	**Objective**	**Number of biomarkers studied**	**Conclusion**	**Quality assessment tool**	**AMSTAR 2**
Fernández-Agra 2022 Ref. [21]	Assess altered salivary biomarkers in patients with BMS	54 salivary biomarkers	Salivary cortisol is the only biomarker that shows a significant increase in BMS.	Newcastle-Ottawa Scale (NOS)	Moderate
Kappes et al., 2023 Ref. [18]	To perform a qualitative and quantitative synthesis of salivary biomarkers in BMS compared with healthy individuals	74 salivary biomarkers, 9 included on the MA	Salivary cortisol and IgA are significantly higher in patients with BMS. Other biomarkers show heterogeneous results.	Newcastle-Ottawa Scale	Low
Kishore et al., 2019 Ref. [17]	To evaluate cytokine expression and its role in the pathophysiology of BMS	8 cytokines	Cytokines appear to be involved in BMS, but the results are heterogeneous and insufficient to identify reliable biomarkers.	Newcastle-Ottawa Scale	Moderate
He et al., 2024 Ref. [20]	To assess whether salivary and serum biomarkers can be used to detect psychological disorders (anxiety, depression) in patients with BMS	43 salivary biomarkers and 35 serum biomarkers	Salivary cortisol is significantly higher in patients with BMS and may be a useful biomarker for assessing psychological status.	Newcastle-Ottawa Scale	Low
Porporatti et al., 2023 Ref. [23]	Is there an association between BMS and stress compared with healthy individuals, and which biomarkers differ?	13 biomarkers	Stress levels and the biomarkers cortisol, α-amylase, IgA, and IL-8 were significantly higher in the BMS group than in the control group.	JBI	Moderate
Brauwers et al., 2024 Ref. [24]	To examine sex hormone variations in patients with BMS and their possible correlation with symptoms and quality of life	4 sex hormones reported overall: oestradiol, DHEA, progesterone and FSH; only oestradiol, DHEA and progesterone contributed salivary evidence, whereas FSH was serum/blood-based	Some hormones (particularly estradiol and FSH) are altered in BMS, but no clear correlation with symptom severity has been observed.	Newcastle-Ottawa Scale	Moderate

The “Quality assessment tool” column refers to the tool used by the original review authors to assess primary studies. The “AMSTAR 2” column refers to the confidence rating assigned in the present umbrella review

**Table 3:** Summary of salivary biomarker evidence in BMS

Biomarker family	Salivary biomarkers	Reviews contributing evidence	Direction of association in BMS	Consistency	Main limitations	Interpretation
Stress-related neuroendocrine	Cortisol	4	↑ Higher in BMS	High	High primary-study overlap; circadian variation; differences in sampling time, saliva type and assay methods	Most consistent salivary signal; likely reflects HPA-axis/stress dysregulation rather than BMS-specific diagnosis
Stress-related autonomic	α-amylase	4	↑ Generally higher in BMS	Moderate	Different measurement units; activity vs. concentration assays; heterogeneous saliva collection; limited comparability across meta-analyses	Promising autonomic/stress-related marker, but still limited by methodological heterogeneity
Immune mucosal stress response	IgA	4	↑ Generally higher in BMS	Moderate	Few primary studies; overlap of evidence; uncertain specificity; possible influence of oral immune status and salivary flow	An exploratory but recurrent marker of mucosal immune or stress-related response
Inflammatory cytokines and immune mediators	IL-2, IL-6, IL-8, IL-10, TNF-α, IL-1β, IL-18, MIP-4, MPO	5	Mixed: ↑, ↓ or no difference	Low	Small samples; different fluids in source reviews; assay variability; inconsistent BMS definitions; sparse cytokine-specific replication	No reproducible salivary cytokine signature is currently supported; IL-8 and IL-18 remain exploratory signals
Endocrine sex steroid markers	DHEA, oestradiol, progesterone, other salivary steroid hormones	5*	Mixed/contradictory	Low	Few salivary studies; menopausal status; hormone therapy; sampling time; many steroid markers below detection limits; serum FSH excluded	Biologically plausible but not yet reliable as salivary biomarkers
Pain-related salivary biomarker	Opiorphin	4	Mixed / no consistent difference	Low	BMS-specific opiorphin data extracted from broader salivary/stress reviews; dedicated opiorphin/orofacial-conditions meta-analysis excluded; sparse evidence and heterogeneous saliva types/assays	Potential pain/homeostatic marker, but not currently supported as a reproducible BMS-specific salivary biomarker
Pain neuropeptide-related	Substance P, NGF, tryptase, calprotectin	2	Mixed or isolated findings	Low	Mostly single-study signals; heterogeneous assays; sparse replication across reviews	Exploratory only
Minerals trace elements	Mg, Ca, Cu, Zn, K, Na, P, B, Cr, Li, Ni, Pb, Sr and others	3	Mg ↓ in some syntheses; others mixed or unchanged	Low to moderate	Analytical heterogeneity; unit differences; dietary/systemic confounding; limited replication for many elements	Insufficient evidence for diagnostic use
Oxidative stress-related	Uric acid, FRAP, nitric oxide-related markers, ROS, nitrites, nitrates, TAC, protein oxidation markers	3	Mixed	Low	Few studies, heterogeneous assays, inconsistent reporting and overlap with inflammatory-marker studies	Mechanistically interesting but preliminary

*BMS *Burning Mouth Syndrome, *CGRP* Calcitonin gene-related peptide, *DHEA* Dehydroepiandrosterone, *FRAP* Ferric reducing ability of plasma, *HPA* Hypothalamic–pituitary–adrenal, *IgA* Immunoglobulin A, *IL* Interleukin, *MIP-4* Macrophage inflammatory protein-4, *MPO* Myeloperoxidase, *NGF* Nerve growth factor, *ROS* Reactive oxygen species, *TAC* Total antioxidant capacity, *TNF-α* Tumour necrosis factor alpha

↑ indicates higher salivary levels in BMS compared with controls; ↓ indicates lower salivary levels; mixed indicates inconsistent direction across studies or reviews. The number of contributing reviews refers only to reviews with extractable evidence of salivary biomarkers for that biomarker family. Serum, plasma, blood, urine, tear and questionnaire-only findings were not counted. FSH was not included as a salivary biomarker because the FSH evidence in Brauwers et al. was serum/blood-based. ^*^Evidence derived from one dedicated sex-hormone review and broader salivary/stress biomarker reviews

### Methodological quality of included reviews

Using AMSTAR 2, four reviews were rated as moderate confidence and two as low confidence. No review achieved a high confidence rating, and none was rated as critically low. The main limitations concerned incomplete reporting of funding sources for primary studies, limited consideration of the impact of primary-study risk of bias on pooled estimates, absence or limited assessment of publication bias in some meta-analyses, and heterogeneous methods across primary studies. Domain-level judgements and overall confidence ratings are presented in Supplementary Table S2.

### Primary-study overlap across reviews

Primary-study overlap was assessed across the six included systematic reviews to avoid interpreting repeated inclusion of the same primary evidence as independent confirmation. Because this umbrella review was restricted to salivary biomarkers, only primary studies reporting extractable salivary biomarker data in BMS were included in the overlap matrix. For mixed reviews, studies reporting only serum, plasma, blood, urine or questionnaire-only outcomes were excluded from the overlap calculation. Across the six included reviews, 34 unique primary studies contributed salivary biomarker evidence. The total number of primary-study occurrences across reviews was 79. The corrected covered area (CCA) was therefore 26.5%: CCA = (79 − 34) / (34 × 6 − 34) = 45 / 170 = 0.265. According to established thresholds, this represents very high overlap. The overlap was mainly driven by the broad salivary biomarker reviews, particularly Kappes et al. [18] and Fernández-Agra et al. [21]; all 17 primary studies included in Fernández-Agra et al. [21] were also represented in Kappes et al. [18]. Fernández-Agra et al. [21] included 17 case-control studies evaluating salivary biomarkers in BMS, while Kappes et al. [18] included 29 studies in the qualitative synthesis and 15 in the quantitative synthesis. Pairwise overlap was also very high between Fernández-Agra et al. [21] and He et al. [20], between Kappes et al. [18] and He et al. [20], and between Kappes et al. [18] and Porporatti et al. [19]. He et al. [20] included both salivary and serum biomarkers, but only the salivary or saliva-containing studies were retained for this salivary-only overlap assessment. Kishore et al. [17] included eight cytokine studies overall, but only the salivary cytokine studies were counted in the present matrix. Brauwers et al. [24] included four sex-hormone studies, of which three assessed salivary hormones and one assessed serum/blood; only the three salivary studies were retained. These findings indicate that agreement across reviews, particularly for cortisol, α-amylase, IgA, DHEA, selected cytokines and opiorphin, should not be interpreted as fully independent replication. Findings repeatedly reported across highly overlapping reviews were therefore interpreted cautiously, with greater weight placed on review scope, methodological quality, recency, salivary specificity and consistency of biomarker direction.

### Evidence synthesis by salivary biomarker family

#### Stress-related and neuroendocrine markers

Cortisol was the most consistently reported salivary biomarker across the included reviews. Fernández-Agra et al. [21] found that salivary cortisol was significantly higher in patients with BMS than in controls and was the only biomarker suitable for meta-analysis in that review. Kappes et al. [18] also reported significantly increased salivary cortisol concentrations in BMS, with low statistical heterogeneity. He et al. [20], in a review focused on biomarkers and psychological disorders, similarly found significantly higher salivary cortisol levels in BMS patients. Porporatti et al. [19], in a stress-focused meta-analysis, also found higher salivary cortisol levels in BMS compared with controls. Regarding α-amylase, it was also repeatedly reported as increased in BMS. Broad reviews of salivary biomarkers and a stress-focused meta-analysis supported this association. However, its interpretation remains limited by methodological heterogeneity, differences in measurement units, activity versus concentration assays, and variability in saliva collection procedures. IgA was reported as higher in several reviews, including the stress-focused meta-analysis. However, its interpretation remains uncertain because IgA may reflect mucosal immune activation, salivary flow, oral immune status or stress-related responses rather than a BMS-specific pathway. Overall, cortisol, α-amylase and IgA appear to reflect stress-related neuroendocrine, autonomic and mucosal immune dysregulation rather than disease-specific diagnostic markers.

#### Inflammatory and immune-related markers

Evidence for salivary inflammatory markers was inconsistent. Reviews addressing cytokines reported heterogeneous findings for IL-2, IL-6, TNF-α, IL-8 and related inflammatory mediators. Some primary studies reported higher levels of selected cytokines in BMS, whereas others reported lower or comparable levels compared with controls. This inconsistency may reflect differences in sample type, assay sensitivity, BMS case definition, comorbid psychological status and small sample sizes. Therefore, current evidence does not support a reproducible salivary cytokine signature for BMS. Inflammatory markers should be interpreted as mechanistic or exploratory biomarkers rather than clinically applicable diagnostic markers.

#### Sex hormones and endocrine markers

Salivary sex hormone findings were also inconsistent. DHEA was the most frequently assessed salivary endocrine marker, but results differed across studies and reviews. Salivary estradiol and progesterone were evaluated in fewer primary studies, with contradictory or non-significant findings. Although hormonal dysregulation is biologically plausible given the predominance of BMS in peri and postmenopausal women, the available salivary evidence does not establish a consistent endocrine biomarker profile. Non-salivary hormonal findings, including serum-based results, were not included in the present synthesis because this umbrella review was restricted to salivary biomarkers.

#### Pain-related salivary biomarkers

Opiorphin was identified as a pain-related salivary biomarker in some of the included reviews, but the available BMS-specific evidence was sparse and inconsistent. Importantly, the dedicated meta-analysis on opiorphin across heterogeneous orofacial conditions was excluded from this umbrella review because it was not focused on salivary biomarkers in BMS as the primary target condition. Therefore, opiorphin was interpreted solely based on BMS-relevant data extracted from the included reviews. Within the included stress-focused meta-analysis, no significant difference in salivary opiorphin concentration was found between BMS patients and controls. Differences in saliva type, assay method, comparator group and the small number of BMS-specific primary studies limit interpretation. Other pain-related biomarkers, including substance P, nerve growth factor, tryptase, calprotectin and CGRP, were reported in isolated studies or reviews and should be considered exploratory. At present, no pain-related salivary biomarker has sufficient reproducibility for diagnostic use in BMS.

#### Trace elements, minerals and oxidative stress-related markers

Trace elements, minerals and oxidative stress-related markers were widely reported but showed limited consistency. Magnesium was reported as decreased in some quantitative syntheses, whereas calcium, copper, zinc and potassium generally showed no consistent or clinically interpretable differences. Oxidative stress-related markers were heterogeneous and were often assessed in single studies. Overall, these biomarkers remain exploratory and are currently limited by small sample sizes, differing analytical methods, a lack of standardized saliva collection protocols, and inconsistent reporting of units.

### Summary of clinically relevant findings

In summary, among the salivary biomarkers assessed across the included reviews, cortisol showed the most consistent evidence of elevation in BMS patients compared with controls. α-amylase and IgA were also repeatedly increased, particularly in reviews of stress-related biomarkers, but their interpretation was limited by methodological heterogeneity, primary-study overlap, and limited disease specificity. DHEA, opiorphin, cytokines, trace elements and oxidative stress-related markers showed inconsistent or sparse evidence. Overall, the current salivary biomarker literature supports the presence of stress-related neuroendocrine, autonomic and mucosal immune dysregulation in BMS, but does not yet support the clinical use of any salivary biomarker as a standalone diagnostic test.

## Discussion

This umbrella review synthesized evidence from six systematic reviews and meta-analyses on salivary biomarkers in BMS. The main finding is that salivary cortisol shows the most consistent evidence of elevation in BMS patients compared with controls, while α-amylase and IgA are also recurrently increased, particularly in reviews addressing stress-related biomarkers. However, the broader salivary biomarker literature remains limited by heterogeneous saliva collection protocols, inconsistent analytical methods, small primary study samples and substantial overlap among primary studies across reviews. Therefore, the current evidence is more consistent with stress-related neuroendocrine, autonomic and mucosal immune dysregulation than with a disease-specific diagnostic biomarker profile.

The very high global CCA indicates that the available review-level evidence is not composed of independent bodies of primary research. Therefore, repeated identification of cortisol, α-amylase, IgA, DHEA, opiorphin or selected cytokines across reviews should be interpreted as convergence within a highly overlapping evidence base, rather than as independent replication across multiple unrelated datasets. This is particularly relevant for stress-related biomarkers, as the same salivary cortisol and α-amylase studies were repeatedly cited in broad salivary biomarker reviews and the stress-focused meta-analysis.

### Methodological quality and evidence integrity

Our AMSTAR 2 appraisal showed variable methodological confidence across the included reviews: four were rated as moderate confidence and two as low confidence. No review achieved a high-confidence rating or was rated critically low. The main limitations concerned incomplete reporting of funding sources for the primary studies, incomplete or variable justification of excluded studies, limited assessment of publication bias or small-study effects in some meta-analyses, and inconsistent incorporation of primary-study risk of bias into the interpretation of findings.

These limitations are important because the apparent convergence around cortisol, α-amylase, and IgA is based on reviews that include overlapping primary studies and heterogeneous methods. For example, Fernández-Agra et al. [21] provided a focused synthesis of salivary biomarker studies and found cortisol to be significantly elevated in BMS, but the included primary studies differed in saliva collection timing, stimulation status and assay procedures. Kappes et al. [18] broadened the scope of salivary biomarkers and identified 74 salivary biomarkers, but only a small subset could be meta-analyzed due to heterogeneity in reporting units and analytical methods. Kishore et al. [17] provided a focused review of cytokine synthesis, but the findings were inconsistent across cytokines, and the review had a narrower scope and an earlier search period. He et al. [20] addressed biomarkers in relation to psychological disorders, but their inclusion of both saliva and serum required careful restriction of the present umbrella synthesis to salivary findings only. Porporatti et al. [19] contributed important evidence on stress-related biomarkers, but interpretation remains constrained by overlap with previous salivary studies and by limited publication-bias assessment in small pooled comparisons. Brauwers et al. [24] specifically addressed sex hormones, but only three of the four included primary studies assessed salivary hormones; the FSH finding was serum/blood-based and was not included in the salivary synthesis.

### Biomarker candidates: signal vs. noise

The synthesized data suggest that BMS is unlikely to have a single universal salivary biomarker profile. Instead, the available evidence points to partially overlapping biological domains, including stress-related neuroendocrine activation, autonomic dysregulation, mucosal immune response, pain modulation, endocrine variation and inflammatory signaling. Among these domains, cortisol showed the most consistent elevation across reviews. α-amylase and IgA were also recurrently increased, particularly in the stress-focused meta-analysis, but both remain limited by methodological heterogeneity and limited disease specificity [21, 23, 24]. In contrast, cytokines, sex hormones, opiorphin, trace elements and oxidative stress-related markers showed inconsistent, sparse or highly overlapping evidence. Therefore, the current evidence supports the use of these biomarkers mainly as research tools for phenotyping and mechanistic exploration, rather than as clinically validated diagnostic tests.

In contrast, findings regarding cytokines (IL-6, IL-1β, TNF-α) and minerals (Mg, Zn, Cu) were highly heterogeneous [17, 18]. One systematic review reported elevated IL-8 based on limited data with contradictory effect sizes, a finding that warrants caution given the small sample size [23]. The sex-hormone review identified inconsistent salivary findings for estradiol, DHEA and progesterone. Although FSH was reported as altered in one included primary study, that finding was serum/blood-based and was therefore not considered part of the salivary biomarker synthesis [24]. The widespread variability in inflammatory and oxidative markers supports the hypothesis that neuroinflammation in BMS may be localized or neurogenic, rather than systemic, or that existing assays lack the sensitivity to detect subtle neuroimmune shifts.

### The biomarker paradox and future directions

A key overarching finding is that methodological inconsistency is pervasive across BMS biomarker research, limiting the certainty of any individual biomarker signal. Only one included review [23] searched grey-literature sources extensively, which limits confidence that unpublished or less accessible evidence was consistently captured across the review-level literature. Together with limited formal assessment of publication bias in some meta-analyses, this reduces certainty in the apparent consistency of some biomarker signals. Furthermore, the lack of funding disclosure in primary studies included in these reviews limits the ability to assess whether funding sources may have influenced primary-study design, assay selection or reporting.

For clinical practice, current evidence supports the utility of salivary cortisol and salivary alpha-amylase as objective state markers of chronic distress, potentially useful for objectively characterizing stress-related biological dysregulation in selected BMS patients, but insufficient for primary diagnosis or treatment monitoring without further validation. For research, this review supports the need for a more standardized research framework: future studies must prioritize (1) prospective registration of protocols, (2) standardization of saliva collection (e.g., fixed circadian timing, unstimulated vs. stimulated distinction), (3) harmonization of assay units to facilitate meta-analysis, and (4) stratification of patients by clinical endotypes (e.g., peripheral neuropathic vs. central/psychogenic) rather than treating BMS as a monolithic condition.

## Conclusions

Current evidence suggests that salivary cortisol and α-amylase are the most consistently altered salivary biomarkers in BMS, probably reflecting stress-related neuroendocrine dysregulation rather than disease-specific diagnostic markers. Evidence for salivary cytokines, sex hormones, opiorphin, trace elements and oxidative stress markers remains heterogeneous and insufficient for clinical diagnostic use. Moving forward, the field requires rigorous, standardized, and large-scale collaborative studies to transform these candidate markers into validated clinical tools. Until then, salivary analysis in BMS remains a valuable research frontier rather than a standalone diagnostic modality.

## Supplementary Information

Supplementary Material 1.

Supplementary Material 2.

## Data Availability

All data analyzed during this study are included in the published systematic reviews that were analyzed in this umbrella review.

## Abbreviations

BMS: Burning Mouth Syndrome
PRISMA: Preferred Reporting Items for Systematic Reviews and Meta-Analyses
