# Supplementary material for: Salivary biomarkers in burning mouth syndrome: an umbrella review of systematic reviews
Source: BMC Oral Health. 2026 May 20;26:1246. doi: 10.1186/s12903-026-08585-z (PMC13361087; doi:10.1186/s12903-026-08585-z)
Supplement: Supplementary file 1 — Supplementary Material 1. [file 12903_2026_8585_MOESM1_ESM.docx]

**Supplementary file**

**Table S1 |** Database-specific search strategies used to identify systematic reviews and meta-analyses on salivary biomarkers in Burning Mouth Syndrome

| Database/search engine | Search field/mode | Search algorithm |
| --- | --- | --- |
| PubMed/MEDLINE | Advanced search; MeSH and Title/Abstract fields | (("Burning Mouth Syndrome"[MeSH Terms] OR "burning mouth syndrome"[Title/Abstract] OR "burning mouth"[Title/Abstract] OR stomatodynia[Title/Abstract] OR glossodynia[Title/Abstract] OR glossalgia[Title/Abstract] OR glossopyrosis[Title/Abstract] OR "burning tongue"[Title/Abstract]) AND ("Saliva"[MeSH Terms] OR saliva[Title/Abstract] OR salivary[Title/Abstract] OR "salivary biomarker*"[Title/Abstract] OR biomarker*[Title/Abstract] OR cortisol[Title/Abstract] OR "alpha-amylase"[Title/Abstract] OR "α-amylase"[Title/Abstract] OR IgA[Title/Abstract] OR cytokine*[Title/Abstract] OR interleukin*[Title/Abstract] OR hormone*[Title/Abstract] OR opiorphin[Title/Abstract] OR "trace element*"[Title/Abstract] OR "oxidative stress"[Title/Abstract]) AND ("systematic review"[Publication Type] OR "meta-analysis"[Publication Type] OR "systematic review"[Title/Abstract] OR "meta-analysis"[Title/Abstract] OR metaanalysis[Title/Abstract] OR "umbrella review"[Title/Abstract] OR "overview of reviews"[Title/Abstract])) |
| Embase | Advanced search; Emtree and title/abstract/keyword fields | ('burning mouth syndrome'/exp OR 'burning mouth syndrome':ti,ab,kw OR 'burning mouth':ti,ab,kw OR stomatodynia:ti,ab,kw OR glossodynia:ti,ab,kw OR glossalgia:ti,ab,kw OR glossopyrosis:ti,ab,kw OR 'burning tongue':ti,ab,kw) AND ('saliva'/exp OR saliva:ti,ab,kw OR salivary:ti,ab,kw OR 'salivary biomarker*':ti,ab,kw OR biomarker*:ti,ab,kw OR cortisol:ti,ab,kw OR 'alpha amylase':ti,ab,kw OR 'alpha-amylase':ti,ab,kw OR 'α-amylase':ti,ab,kw OR iga:ti,ab,kw OR cytokine*:ti,ab,kw OR interleukin*:ti,ab,kw OR hormone*:ti,ab,kw OR opiorphin:ti,ab,kw OR 'trace element*':ti,ab,kw OR 'oxidative stress':ti,ab,kw) AND ('systematic review'/exp OR 'meta analysis'/exp OR 'systematic review':ti,ab,kw OR 'meta-analysis':ti,ab,kw OR metaanalysis:ti,ab,kw OR 'umbrella review':ti,ab,kw OR 'overview of reviews':ti,ab,kw) |
| Web of Science Core Collection | Advanced search; Topic field | TS=(("burning mouth syndrome" OR "burning mouth" OR stomatodynia OR glossodynia OR glossalgia OR glossopyrosis OR "burning tongue") AND (saliva OR salivary OR "salivary biomarker*" OR biomarker* OR cortisol OR "alpha-amylase" OR "alpha amylase" OR "α-amylase" OR IgA OR cytokine* OR interleukin* OR hormone* OR opiorphin OR "trace element*" OR "oxidative stress") AND ("systematic review" OR "meta-analysis" OR metaanalysis OR "umbrella review" OR "overview of reviews")) |
| Scopus | Advanced search: Title, Abstract and Keywords | TITLE-ABS-KEY(("burning mouth syndrome" OR "burning mouth" OR stomatodynia OR glossodynia OR glossalgia OR glossopyrosis OR "burning tongue") AND (saliva OR salivary OR "salivary biomarker*" OR biomarker* OR cortisol OR "alpha-amylase" OR "alpha amylase" OR "α-amylase" OR IgA OR cytokine* OR interleukin* OR hormone* OR opiorphin OR "trace element*" OR "oxidative stress") AND ("systematic review" OR "meta-analysis" OR metaanalysis OR "umbrella review" OR "overview of reviews")) |
| TRIP Database | Basic or advanced search; Boolean phrase search | ("burning mouth syndrome" OR stomatodynia OR glossodynia OR glossalgia OR "burning tongue") AND (saliva OR salivary OR "salivary biomarkers" OR biomarkers OR cortisol OR "alpha-amylase" OR IgA OR cytokines OR interleukins OR hormones OR opiorphin OR "trace elements" OR "oxidative stress") AND ("systematic review" OR "meta-analysis" OR "umbrella review") |
| Google Scholar | Advanced search/phrase search; first 200 results screened, sorted by relevance | ("burning mouth syndrome" OR stomatodynia OR glossodynia OR glossalgia OR "burning tongue") ("saliva" OR "salivary biomarkers" OR cortisol OR "alpha-amylase" OR IgA OR cytokines OR hormones OR opiorphin) ("systematic review" OR "meta-analysis") |

**Table note:** Searches were conducted without date or language restrictions. Search syntax was adapted to the requirements of each database or search engine. PubMed/MEDLINE was searched using MeSH and Title/Abstract fields; Embase was searched using Emtree and title/abstract/keyword fields; Web of Science used the Topic field; Scopus used TITLE-ABS-KEY; TRIP and Google Scholar were searched using simplified Boolean-compatible strings. Google Scholar results were screened in order of relevance because the platform does not support the same level of reproducible field-controlled searching as bibliographic databases.

**Table S2 |** Methodological appraisal and confidence in the results of the included systematic reviews using AMSTAR 2.

| Author / Year | 1 | 2 | 3 | 4 | 5 | 6 | 7 | 8 | 9 | 10 | 11 | 12 | 13 | 14 | 15 | 16 | Overall AMSTAR 2 rating |
| --- | --- | --- | --- | --- | --- | --- | --- | --- | --- | --- | --- | --- | --- | --- | --- | --- | --- |
| Fernández-Agra 2022  Ref. 21 | Yes | Yes | Yes | Yes | Yes | Yes | Yes | Yes | Yes | No | Partially yes | No | Yes | Yes | Yes | Yes | Moderate |
| Kappes et al., 2023  Ref. 18 | Yes | Yes | Yes | Yes | Yes | Yes | Partially yes | Yes | Yes | No | Partially yes | No | Yes | No | No | Yes | Low |
| Kishore et al., 2019  Ref. 17 | Yes | Yes | Yes | Yes | Yes | Yes | Yes | Yes | Yes | No | N/A | N/A | Yes | No | N/A | Yes | Moderate |
| He et al., 2024  Ref. 20 | Yes | Yes | Yes | Yes | Yes | Yes | Yes | Yes | Yes | No | Partially yes | No | Yes | No | No | Yes | Low |
| Porporatti et al., 2023  Ref. 25 | Yes | Yes | Yes | Yes | Yes | Yes | Yes | Yes | Yes | No | Yes | No | Yes | Yes | Partially yes | Yes | Moderate |
| Brauwers et al., 2024  Ref. 26 | Yes | Yes | Yes | Yes | Yes | Yes | Yes | Yes | Yes | No | N/A | N/A | Yes | No | N/A | Yes | Moderate |

## Columns 1–16 correspond to the 16 AMSTAR 2 methodological domains evaluated; AMSTAR 2 domains: 1, PICO components; 2, protocol registered before commencement; 3, explanation of review design; 4, comprehensive literature search; 5, duplicate study selection; 6, duplicate data extraction; 7, list and justification of excluded studies; 8, description of included studies; 9, satisfactory technique for assessing risk of bias in primary studies; 10, reporting of funding sources for primary studies; 11, appropriate meta-analytical methods; 12, consideration of risk of bias in meta-analysis; 13, consideration of risk of bias when interpreting results; 14, explanation/discussion of heterogeneity; 15, assessment of publication bias; 16, reporting of conflicts of interest. The overall confidence rating in the last column reflects the combined judgment across all 16 domains. N/A, Not Applicable. AMSTAR 2 overall ratings were assigned according to the official AMSTAR 2 guidance and were not calculated as numerical scores. Items 11, 12 and 15 were marked as not applicable for systematic reviews without meta-analysis. For meta-analyses with fewer than 10 studies per pooled comparison, formal publication-bias assessment was considered limited; when authors acknowledged and discussed this limitation, item 15 was judged as partially yes rather than no.

**Table S3 |** Citation matrix for primary-study overlap across included reviews

| Primary study | Fernández-Agra 2022 | Kappes 2023 | He 2024 | Kishore 2019 | Brauwers 2024 | Porporatti 2023 | Occurrences |
| --- | --- | --- | --- | --- | --- | --- | --- |
| Acharya et al., 2019 |  | ✓ |  |  |  |  | 1 |
| Aitken-Saavedra et al., 2021 | ✓ | ✓ |  |  |  | ✓ | 3 |
| Amenábar et al., 2008 | ✓ | ✓ | ✓ |  |  | ✓ | 4 |
| Boras et al., 2010 | ✓ | ✓ |  |  |  |  | 2 |
| Borelli et al., 2010 | ✓ | ✓ |  |  |  |  | 2 |
| Boucher et al., 2016/2017 | ✓ | ✓ | ✓ |  |  | ✓ | 4 |
| Castillo-Felipe et al., 2021 |  |  |  |  |  | ✓ | 1 |
| de Moura et al., 2007 | ✓ | ✓ |  |  |  |  | 2 |
| de Souza et al., 2015 |  | ✓ |  | ✓ |  | ✓ | 3 |
| Dias Fernandes et al., 2009 | ✓ | ✓ | ✓ |  | ✓ |  | 4 |
| Glick et al., 1976 |  | ✓ |  |  |  |  | 1 |
| Henkin et al., 2012 |  | ✓ |  |  |  |  | 1 |
| Hershkovich and Nagler, 2004 |  | ✓ |  |  |  |  | 1 |
| Imura et al., 2016 | ✓ | ✓ |  |  |  | ✓ | 3 |
| Ji et al., 2017 |  | ✓ |  | ✓ |  | ✓ | 3 |
| Kim et al., 2012 | ✓ | ✓ |  |  | ✓ | ✓ | 4 |
| Koike et al., 2014 |  |  |  |  |  | ✓ | 1 |
| Loeb et al., 2008 |  | ✓ |  |  |  |  | 1 |
| Lončar-Brzak et al., 2020 | ✓ | ✓ |  |  | ✓ |  | 3 |
| López-Jornet et al., 2009 |  |  |  |  |  | ✓ | 1 |
| López-Jornet et al., 2014/2015 | ✓ | ✓ | ✓ |  |  |  | 3 |
| López-Jornet et al., 2020 | ✓ | ✓ | ✓ |  |  | ✓ | 4 |
| Moreau et al., 2022 |  |  |  |  |  | ✓ | 1 |
| Nosratzehi et al., 2017 | ✓ | ✓ | ✓ |  |  | ✓ | 4 |
| Pekiner et al., 2009 | ✓ | ✓ | ✓ | ✓ |  |  | 4 |
| Salarić et al., 2017 | ✓ | ✓ |  |  |  | ✓ | 3 |
| Shigeyama-Haruna et al., 2013 |  | ✓ |  |  |  | ✓ | 2 |
| Simčić et al., 2006 |  | ✓ |  | ✓ |  |  | 2 |
| Srinivasan et al., 2008 |  | ✓ |  |  |  |  | 1 |
| Suh et al., 2009 |  | ✓ |  | ✓ |  | ✓ | 3 |
| Tammiala-Salonen and Söderling, 1993 |  | ✓ |  |  |  |  | 1 |
| Tvarijonaviciute et al., 2017 | ✓ | ✓ |  |  |  | ✓ | 3 |
| Zidverc-Trajković et al., 2009 | ✓ | ✓ |  |  |  |  | 2 |
| Škrinjar et al., 2020 |  |  |  |  |  | ✓ | 1 |

**Table note:** Only primary studies contributing extractable salivary biomarker data in BMS were counted. Serum-only, plasma-only, blood-only, urine-only and questionnaire-only studies were excluded from this overlap matrix. Some primary studies are reported with different publication years across reviews due to online-first versus issue publication dates; these were harmonized as the same study when the author group and study content were identical. CCA, corrected covered area.

**Table S4 |** Pairwise overlap between included reviews

| Review pair | Shared primary studies | Unique primary studies in pair | Pairwise CCA (%) | Interpretation |
| --- | --- | --- | --- | --- |
| Fernández-Agra et al. / Kappes et al. | 17 | 29 | 58.6 | Very high |
| Fernández-Agra et al. / He et al. | 7 | 17 | 41.2 | Very high |
| Kappes et al. / Porporatti et al. | 13 | 34 | 38.2 | Very high |
| Fernández-Agra et al. / Porporatti et al. | 9 | 26 | 34.6 | Very high |
| Kappes et al. / He et al. | 7 | 29 | 24.1 | Very high |
| He et al. / Porporatti et al. | 4 | 21 | 19.0 | Very high |
| Fernández-Agra et al. / Brauwers et al. | 3 | 17 | 17.6 | Very high |
| Kappes et al. / Kishore et al. | 5 | 29 | 17.2 | Very high |
| Kishore et al. / Porporatti et al. | 3 | 20 | 15.0 | High |
| He et al. / Brauwers et al. | 1 | 9 | 11.1 | High |
| Kappes et al. / Brauwers et al. | 3 | 29 | 10.3 | High |
| He et al. / Kishore et al. | 1 | 11 | 9.1 | Moderate |
| Brauwers et al. / Porporatti et al. | 1 | 20 | 5.0 | Slight |
| Fernández-Agra et al. / Kishore et al. | 1 | 21 | 4.8 | Slight |
| Kishore et al. / Brauwers et al. | 0 | 8 | 0.0 | Slight |

**Table note:** Pairwise CCA was calculated as the number of shared primary studies divided by the number of unique primary studies in each review pair. Thresholds used: slight overlap, 0–5%; moderate, 6–10%; high, 11–15%; very high, >15%.
